# Contrast-enhanced computed tomography assisted diagnosis of bleeding caused by colonic angiodysplasia: A case report

**DOI:** 10.1097/MD.0000000000039984

**Published:** 2024-10-04

**Authors:** Yinze Chen, Xiaomin Liu, Liang Guo, Ying Tang, Xiangwei Meng

**Affiliations:** a Department of Gastroenterology, The First Hospital of Jilin University, Changchun, China; b Department of Gastroenterology, Henan Province Chest Hospital, Chest Hospital of Zhengzhou University, Zhengzhou, China; c Department of Pathology, The First Hospital of Jilin University, Changchun, China.

**Keywords:** angiodysplasia, bleeding, computed tomography, diagnostic, treatment

## Abstract

**Rationale:**

Angiodysplasia (AD) in the gastrointestinal tract is a degenerative vascular condition characterized by vascular dilation, tortuosity, and arteriovenous connections within the mucosal and submucosal layers. AD is a significant cause of lower gastrointestinal bleeding in the elderly, often presenting as chronic, intermittent hemorrhage. The research challenge lies in the potential for multiple and minute AD lesions to be missed during endoscopy, which may lead to post-hemostatic rebleeding.

**Patient concerns:**

An 82-year-old female with a history of coronary artery disease treated with aspirin, presenting with recurrent melena and anemia. The patient exhibited a suboptimal response to blood transfusions and octreotide therapy.

**Diagnosis:**

After excluding tumorous bleeding in the initial contrast-enhanced computed tomography (CECT), we suspect vascular bleeding as the most probable etiology.

**Interventions:**

The initial colonoscopy was unsuccessful due to the occurrence of ventricular tachycardia. Considering the patient’s advanced age, cardiac dysfunction, and frailty, a repeat CECT were conducted during episodes of suspected active bleeding. Vascular dilatation within the colonic hepatic flexure wall was visualized during the venous phase, accompanied by contrast agent extravasation into the intestinal lumen. The subsequent urgent colonoscopy and pathological specimens from surgical resection supported the diagnosis of colonic AD.

**Outcomes:**

After the surgery, the patient did not experience melena thereafter.

**lessons:**

We highlight that CECT for diagnosing AD-related bleeding offers higher safety and convenience, potentially superior to digital subtraction angiography, particularly in critically ill elderly patients.

## 1. Introduction

Gastrointestinal angiodysplasia (AD) is a vascular malformation that predominantly affects small venules, capillaries, and arterioles within the mucosal and submucosal layers, especially in the older individuals.^[1]^ Vascular degenerative changes, vascular developmental abnormalities, and coagulation disorders may be contributed to the pathogenesis of this disease. Hemorrhage is the primary complication of AD, with patients typically being asymptomatic in the absence of bleeding episodes. AD is commonly found in the proximal small intestine and right colon, serving as a significant cause of lower gastrointestinal bleeding (LGIB) in the elderly population.^[2,3]^ In the demographic undergoing colonoscopy, the detection rate of colonic AD is approximately 0.6% to 0.9%, with active bleeding observed in around 5% to 30% of these cases.^[4,5]^ Endoscopy is the primary diagnostic modality for AD. In cases where endoscopy is unsuccessful or poorly tolerated by patients, angiography serves as a valuable alternative. Compared to digital subtraction angiography (DSA), computed tomography angiography (CTA) offers the advantages of simplified operation, lower dosage of intravenous contrast agent, and avoidance of complications associated with arterial puncture. Prior studies indicate that the diagnostic reliability of colonic AD-induced bleeding using CTA alone is comparable to the combined approach of colonoscopy and DSA.^[6]^ AD-related bleeding is characterized by a slow, intermittent, and recurrent course. Contrast-enhanced computed tomography (CECT) enables imaging across 3 phases, aiding in the localization of slow bleeding sources and identifying the hemorrhage from nonvascular lesions.^[7]^ Despite the clinical utility of CECT, a comprehensive evaluation of its diagnostic reliability in identifying AD-related bleeding remains scarce in the literature. Here, we present a case study of a critically ill patient with LGIB, where colonic AD was successfully detected using repeat CECT following negative initial CECT findings and an incomplete colonoscopy. Our report underscores the safety and practicality of employing CECT in diagnosing severe cases of bleeding caused by AD.

## 2. Case report

An 82-year-old female presented with symptoms of black stools. She had a history of acute myocardial infarction 8 years ago and was currently on a daily oral aspirin regimen of 100 mg. Starting a month ago, she has been passing melena approximately every 3 days, with each episode amounting to around 100 mL. Despite discontinuing aspirin and resting at home for a month, her symptoms persisted, accompanied by fatigue and chest tightness. Initial examination at a primary healthcare facility revealed a hemoglobin level of 67 g/L and a positive fecal occult blood test. However, a CECT failed to identify the source of bleeding. After receiving fasting and blood transfusion therapy, her hemoglobin levels improved to 106 g/L. Following a temporary cessation of melena for 5 days, she resumed oral intake but subsequently relapsed. Subsequent treatments over the following 2 weeks included blood transfusions and octreotide therapy in a fasting state, yielding limited efficacy. Following a significant episode of dark red watery stool (200 mL), she underwent colonoscopy with blood transfusion. However, the procedure was prematurely terminated due to the onset of ventricular tachycardia. The examination ruled out bleeding from the upper gastrointestinal tract, rectum, and sigmoid colon. Subsequently, the patient was transferred to our hospital for further management.

**Figure 1.**
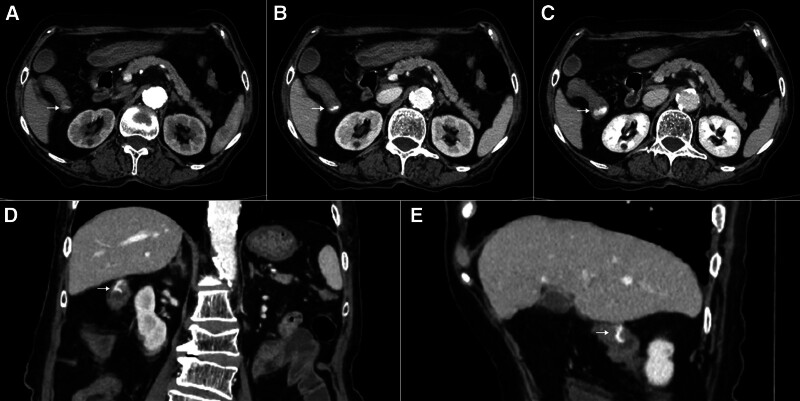
Contrast-enhanced computed tomography manifestations of colonic angiodysplasia. The volume of contrast material within the intestinal lumen increased progressively over time (A–C). The dilated vessels were enhanced within the intestinal wall (D–E). (A) Arterial phase in a horizontal position. (B) Venous phase in a horizontal position. (C) Delayed phase in a horizontal position. (D) Venous phase in a coronal position. (E) Venous phase in a sagittal position.

Additional examinations of the patient revealed a hemoglobin level of 89 g/L and a pro-BNP level of 2230 pg/mL, with normal liver, renal, and coagulation functions. The echocardiogram showed diffuse hypokinesis of the left ventricle and reduced systolic and diastolic function (left ventricular ejection fraction: 43%, E/A: 0.74). As the initial CECT result was negative, we tentatively ruled out tumorous bleeding. Due to the intermittent nature of the bleeding, we suspect that it may be originating from vascular lesions. Due to the patient’s frequent ventricular premature contractions and heart failure, blood transfusions and octreotide therapy with fasting was continued, without undergoing colonoscopy or DSA examination. Melena persisted, occurring once every 1 to 3 days, with each episode approximately 100 mL. The patient received a total of 8.5 units of red blood cell transfusions, with hemoglobin levels ranging from a peak of 99 g/L to a low of 50 g/L. On the 18th day after admission, the patient passed 300 mL of dark red stool within 3 hours, prompting an urgent reexamination of the abdominal CECT. Extravasation of the contrast agent from the hepatic flexure of the colon into the intestinal lumen was observed, with dilated vessels enhancing in the venous phase within the colonic wall. The presence of AD in this location was suspected (Fig. 1).

**Figure 2.**
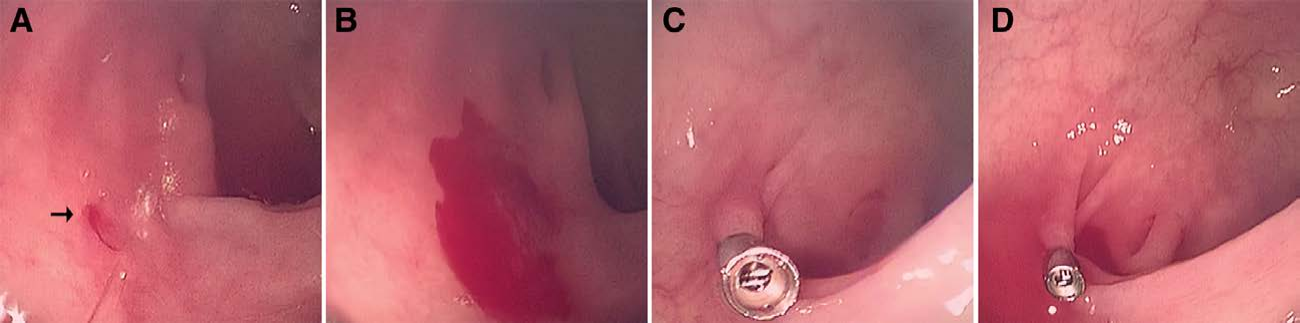
Endoscopic findings of colonic angiodysplasia. (A) A red lesion approximately 2 mm in diameter with active bleeding. (B) Continuous bleeding observed after endoscopic irrigation. (C) Hemostasis attempted with titanium clip closure. (D) Failed hemostasis.

**Figure 3.**
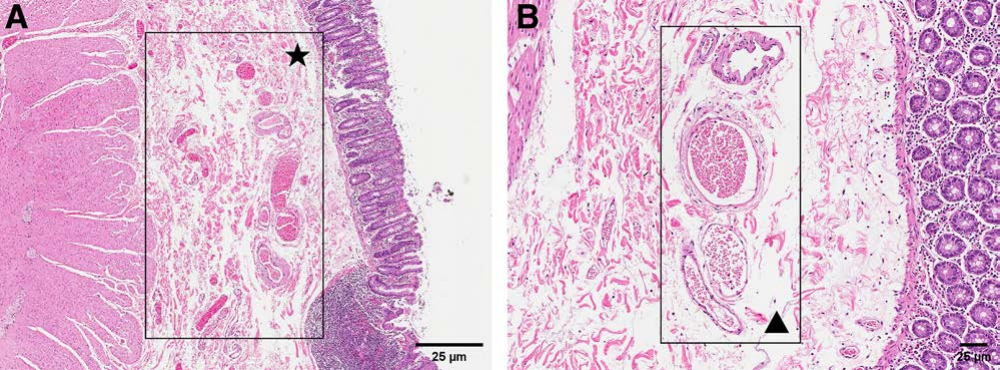
Microscopic examination of resection specimens: (A) Several abnormally dilated vessels can be seen in the edematous submucosa (hematoxylin–eosin, original magnification × 40). (B) Blood stasis can be seen in the lumen of dilated venules, capillaries, and arterioles (hematoxylin–eosin, original magnification × 100).

During an emergency repeat colonoscopy, a bright red spot approximately 2 mm in diameter was identified at the hepatic flexure of the colon, where continuous bleeding was observed despite repeated irrigation. Hemostasis was not achieved after titanium clip closure of the lesion (Fig. 2). To achieve definitive hemostasis, the patient opted for surgical resection of the affected intestinal segment. Postoperative histopathological examination revealed mucosal edema in the specimen, with multiple abnormally dilated small veins, capillaries, and small arteries, along with vascular congestion (Fig. 3). We further confirmed the diagnosis of colonic AD.

There was no recurrence of melena postoperatively. The hemoglobin level was 103 g/L at discharge 2 weeks postoperatively. It increased to 127 g/L at discharge 1 year postoperatively. A telephone follow-up at 1.5 years post-discharge confirmed that the patient had no recurrence of melena.

## 3. Discussion

Gastrointestinal AD is an important cause of LGIB.^[2]^ Chronic, intermittent bleeding is a common clinical presentation, while a minority of patients may also experience acute massive bleeding. Age over 75 years, more than 10 lesions, coronary artery disease, diabetes, and chronic kidney disease are independent risk factors for bleeding in AD.^[1]^ Approximately 70% to 80% of colonic AD lesions that bleed have a diameter of about 1 to 2 mm, making them prone to endoscopic under-recognition.^[4,8]^ More than half of the cases present with multiple lesions, and bleeding may persist at another site even after endoscopic hemostasis.^[5]^ The 30-day readmission rate for AD patients with recurrent bleeding can reach 20%. Improving diagnostic rates and reducing rebleeding rates can help enhance patient outcomes.^[9]^

Vascular degeneration may represent a primary pathogenic mechanism of AD. The periodic contractions of the intestinal smooth muscle lead to recurrent occlusion of the intrinsic muscular layer vessels, resulting in the dilation and distortion of small veins, capillaries, and arterioles in the mucosal and submucosal layers, potentially accompanied by arteriovenous shunting.^[10]^ Dilated and tortuous vessels with arteriovenous connections are predisposed to rupture and bleeding. Abnormal regulation of angiogenesis and impaired coagulation function may also play a role in the pathogenesis. Coronary artery disease, heart failure, and chronic kidney disease can exacerbate chronic hypoxia and reduced blood flow in the gastrointestinal mucosa, leading to an elevation in angiopoietin (Ang) Ang-2 levels.^[11,12]^ A decreased Ang-1/Ang-2 ratio can induce abnormal angiogenesis and reduce vascular stability. Severe aortic valve stenosis-induced high flow shear stress can lead to conformational changes and excessive consumption of high molecular weight von Willebrand factor multimers, resulting in an increased release of Ang-2 and a direct elevation of the risk of bleeding.^[13]^ Direct oral anticoagulant administration can increase the risk of gastrointestinal AD bleeding by 4.16 times, and antiplatelet agents can have a similar effect.^[14]^

Colonoscopy serves as a crucial method for identifying the etiology of LGIB and diagnosing AD. In cases of obscure gastrointestinal bleeding (OGIB), other investigations such as enhanced CT, capsule endoscopy, or enteroscopy are required.^[15]^ Endoscopically, colonic AD manifests as punctate or patchy red lesions, flat or slightly elevated, exhibiting dendritic or spider-like branch vessels at the periphery, typically <5 mm in diameter, devoid of arterial pulsations. Lesions that are small, pale in color, and multifocal are susceptible to diagnostic oversight due to mucosal folds or residual fecal matter obscuring their visibility. Assessment of pulsatility on lesion surfaces aids in discerning arterial hemorrhage, including lesions such as Dieulafoy lesion and arteriovenous malformations.^[16]^ The application of Gel Immersion technique, involving the instillation of clear gel into the intestinal lumen, serves to prevent the admixture of water with fresh blood or feces, improving endoscopic visualization and reducing omission diagnostic rate.^[17]^ argon plasma coagulation (APC) is the preferred therapeutic modality for managing bleeding caused by AD. Given the relatively thin intestinal walls of the right colon and small intestine, the fine output energy of APC in PRECISE mode diminishes the risk of perforation.^[18]^ For anticoagulated patients, the combined approach of APC and titanium clip closure effectively mitigates the risk of recurrent bleeding without escalating thermal injury.^[19]^ Sriram et al implemented endoscopic mucosal resection, involving injection, elevation, thermal snare excision of lesions, and coagulation of submucosal blood vessels, resulting in an absence of posttreatment hemorrhagic events in 6 patients, highlighting the potential of this technique for broader clinical application.^[20]^

In the context of considering vascular bleeding, selective superior mesenteric artery DSA is proposed as an alternative approach to endoscopy. AD manifestations on DSA include dense and turbid dilated vascular shadows, with contrast agent retention observed in the capillary and venous phases.^[10]^ Contrast extravasation serves as a direct criterion for evaluating active bleeding. In cases where endoscopic interventions are ineffective, transcatheter arterial embolization can be considered. Super-selective transcatheter arterial embolization has demonstrated efficacy in reducing the risk of intestinal ischemia and collateral vessel bleeding.^[21]^ CTA and CECT present as noninvasive diagnostic modalities with rapid execution and high-safety profiles, minimizing complications associated with arterial punctures such as bleeding, infection, pseudoaneurysm, and arteriovenous fistula formation. The injected contrast agent dose and radiation dose are also lower than DSA.^[22]^ Junquera et al reported that the sensitivity and specificity of CTA in diagnosing colonic AD-related bleeding were 70.0% and 100%, respectively; the accuracy of CTA alone and colonoscopy combined with DSA for diagnosis was comparable.^[6]^ Multiphase CECT offers an advantage in diagnosing slow and sustained bleeding and nonvascular etiologies of bleeding. Notably, CT diagnostic efficacy is heightened in scenarios of rapid or voluminous bleeding. Conversely, CT diagnostic efficacy may diminish when the bleeding rate falls below 0.3 to 0.5 mL/min or ceases.^[23]^ He et al’s meta-analysis demonstrated that small bowel computed tomography enterography achieved sensitivity, specificity, and ROC values of approximately 0.724, 0.752, and 0.792, respectively, in the diagnosis of OGIB.^[24]^ While CECT serves as a valuable adjunctive tool in the diagnostic workup for OGIB, it may not entirely replace capsule endoscopy. Further investigations are warranted to assess the reliability of CECT in diagnosing colonic AD-related bleeding.

In our case, the patient has a history of coronary artery disease, has been undergoing long-term oral antiplatelet therapy, and is in a state of heart failure. These factors may be associated with the onset of the condition. The characteristics of advanced age and a history of coronary artery disease may contribute to suboptimal responses to blood transfusions and octreotide therapy in patients. Slow bleeding rate or temporary cessation may lead to the initial negative CECT result. During the initial colonoscopy, the patient experienced ventricular tachycardia, leading to procedural failure, which delayed the timely identification of the bleeding etiology, prolonged the patient’s disease course, and diminished the patient’s quality of life. Since CECT temporarily ruled out gastrointestinal tumors, we suspect that the intermittent bleeding may originate from vascular lesions. Considering the compromised cardiac function and frail physical condition of the patients, we repeated the utilization of a high-safety CECT technique to identify dilated vessels within the intestinal wall and extravasated contrast agent, providing supporting evidence for the diagnosis of bleeding due to colonic AD. Hence, we posit that CECT may present superior safety and practicality in diagnosing critical cases of AD-related bleeding compared to DSA examinations.

## 4. Conclusions

Gastrointestinal AD-related bleeding leads to chronic blood loss that significantly impairs patients’ quality of life and escalates mortality risks. Colonoscopy stands out as the preferred diagnostic modality for identifying the etiology of LGIB and diagnosing AD. For critically ill patients suspected of vascular bleeding and with contraindications to endoscopy, the convenience and safety of CECT may be preferable to DSA. Future research endeavors could delve deeper into exploring the diagnostic utility of CECT in AD cases, furnishing additional insights to guide the management of critically ill patients experiencing bleeding caused by AD.

## Author contributions

**Data curation:** Yinze Chen, Xiaomin Liu.

**Formal analysis:** Yinze Chen, Xiaomin Liu.

**Funding acquisition:** Ying Tang.

**Investigation:** Yinze Chen.

**Project administration:** Xiangwei Meng.

**Resources:** Liang Guo.

**Writing – original draft:** Yinze Chen, Xiaomin Liu.

**Writing – review & editing:** Xiangwei Meng, Ying Tang.
